# Temporal Production Signals in Parietal Cortex

**DOI:** 10.1371/journal.pbio.1001413

**Published:** 2012-10-30

**Authors:** Blaine A. Schneider, Geoffrey M. Ghose

**Affiliations:** Department of Neuroscience, Center for Magnetic Resonance Research, University of Minnesota, Minneapolis, Minnesota, United States of America; McGill University, Canada

## Abstract

We often perform movements and actions on the basis of internal motivations and without any explicit instructions or cues. One common example of such behaviors is our ability to initiate movements solely on the basis of an internally generated sense of the passage of time. In order to isolate the neuronal signals responsible for such timed behaviors, we devised a task that requires nonhuman primates to move their eyes consistently at regular time intervals in the absence of any external stimulus events and without an immediate expectation of reward. Despite the lack of sensory information, we found that animals were remarkably precise and consistent in timed behaviors, with standard deviations on the order of 100 ms. To examine the potential neural basis of this precision, we recorded from single neurons in the lateral intraparietal area (LIP), which has been implicated in the planning and execution of eye movements. In contrast to previous studies that observed a build-up of activity associated with the passage of time, we found that LIP activity decreased at a constant rate between timed movements. Moreover, the magnitude of activity was predictive of the timing of the impending movement. Interestingly, this relationship depended on eye movement direction: activity was negatively correlated with timing when the upcoming saccade was toward the neuron's response field and positively correlated when the upcoming saccade was directed away from the response field. This suggests that LIP activity encodes timed movements in a push-pull manner by signaling for both saccade initiation towards one target and prolonged fixation for the other target. Thus timed movements in this task appear to reflect the competition between local populations of task relevant neurons rather than a global timing signal.

## Abstract 2

Neuronal activity in the lateral intraparietal cortex of monkeys reflects the passage of time and is sufficiently precise to explain timed eye movements.

## Author Summary

One advantage of studying sensory systems is that external stimuli can be readily controlled and quantified. Our internal perception of time, however, is not as easily approachable. To address this challenge, we developed a task that leverages our knowledge of the neuronal circuits underlying eye movements. Monkeys were trained to move their eyes consistently at regular time intervals without any external cueing or immediate expectation of reward. The animals were remarkably precise and consistent in their timing. Recordings from individual lateral intraparietal area (LIP) neurons, an area associated with eye movement planning, showed a linear decrease in activity between eye movements. This contrasts with previous studies that have reported increases in LIP activity immediately prior to eye movements and suggests that expectation of reward may have influenced such results. We also found that variations in this decreasing activity were predictive of the animals’ timing. Finally, we demonstrate through a simple model that LIP activity was sufficiently precise to completely explain the timing of the animals' actions. The model demonstrates that a precise internal sense of time can arise by comparing activity across different neuronal populations.

## Introduction

In order to plan for upcoming movements and actions, the brain must be able to represent the passage of time. However, the nature of signals that encode time (measurement) and the way in which these signals are utilized in order to produce movement (production) are unclear [1]–[3]. In particular, signals associated with temporal measurement, the representation of the passage of time between external events, need not reflect temporal production, the execution of a behavior at a specific time [3]–[8]. Although the passage of time must be monitored during both temporal measurement and temporal production, in temporal measurement consistencies in the sequence and timing of external events can serve as a clock. For example, we can decide that it is time to go home after a workday by looking at the clock (temporal measurement) or by an internal judgment (temporal production), irrespective of external cues, that it is getting late.

The distinction is particularly important when looking for the physiological basis of temporal representations because consistent variations in activity over time in a stereotyped task may reflect task parameters, such as stimulus events or probabilities, that systematically vary over time, rather than representing time itself [9]–[21]. For example, if the animal is explicitly cued when to make a movement and that cue tends to happen at certain times, then activity may represent the time-dependent probability of cueing rather than temporal production per se [16]. Similarly, if movements are linked to sensory events, such as the approach of a moving target [22], temporal variations in activity may reflect some combination of stimulus and task dynamics rather than the movement itself.

Activity could also reflect external events such as reward that, by virtue of being tightly coupled to the upcoming movement, can be readily anticipated. In most timing studies, rewards are contingent on a single specific eye movement [4],[9],[13],[16],[18],[20] that is cued at a particular time. Therefore, both the sensory cue instructing the movement and the reward that is linked to the production of that movement can be readily anticipated. This is of particular interest because neurons within the parietal cortex have been shown to modulate their activity during visual anticipation [23] and reward anticipation [24]–[29] as well as during movement planning [9],.

**Figure 1:**
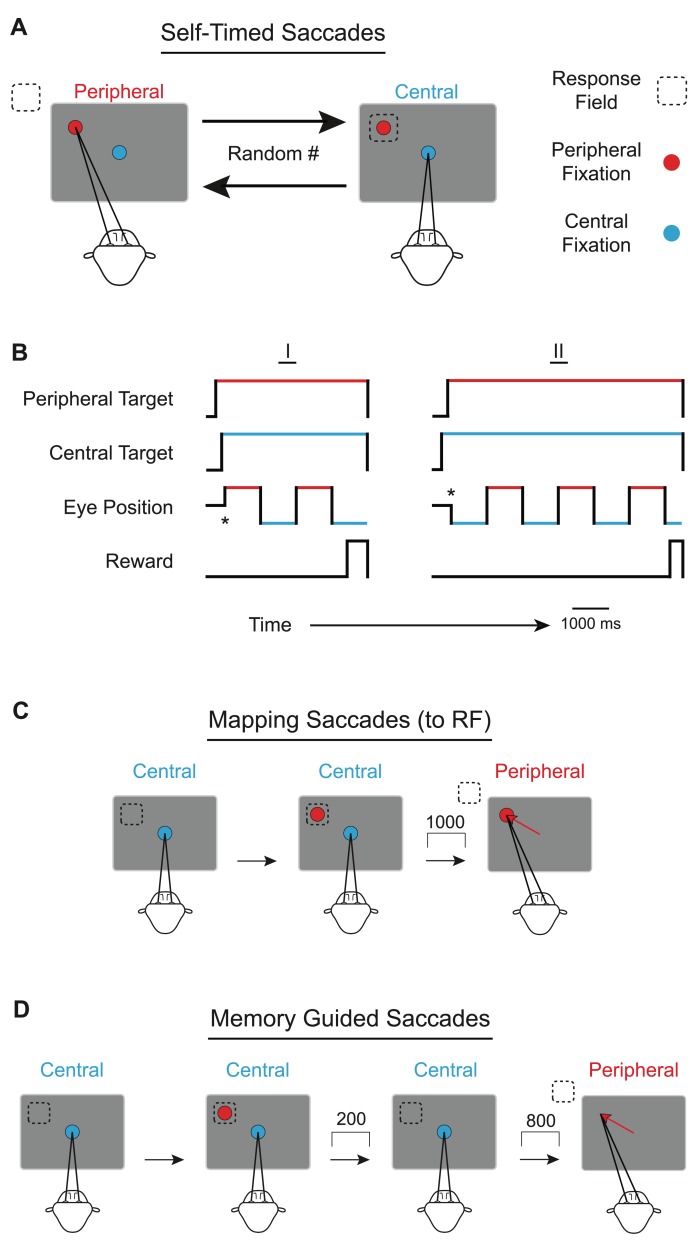
Self-timed rhythmic-saccade task (A) with example trials (B), the mapping task (C), and the memory-guided delayed-saccade task (D). (A) For the self-timed task, animals were required to fixate on the first of the two targets to appear (randomly) on a computer monitor. One target was located near the center of the screen (blue), while the other target was located peripherally (red). Immediately following fixation of the first target, the second target appeared. The animal was then required to saccade back and forth between the two fixed targets at a 1 s interval (allowable error window = ±200 ms) for a random number of saccades in order to receive a juice reward. When the animal fixated the central target, the response field (RF) of the recorded neuron was located at the peripheral target. (B) In these example trials, the fixation of the first target is indicated with an asterisk. Following initial fixation (*), the animal was required to produce three saccades prior to receiving a reward in the first example (I) and six saccades prior to receiving a reward in the second example (II). Notice that once the targets appeared, both targets remained constantly displayed so that no visual cues were provided to the animal. Additionally, trials may also end at any time, regardless of when saccades occur. (C) In the mapping task, the peripheral target appears immediately following fixation of the central target. One second later, the fixation point turns off and the animal saccades to the peripheral target. In this task, only a single saccade is required to receive a reward. Although mapping targets are placed at various points about the monitor, the figure shows an example where the target is placed within the RF. (D) For the memory-guided delayed-saccade task, animals first fixate the central target. Following fixation, the peripheral target appears in the RF for 200 ms. The peripheral target then turns off and the animal is required to remember the peripheral target location. Eight hundred milliseconds after the peripheral target is extinguished, the central fixation point is also extinguished, cueing the animal to make a saccade to the remembered location in order to receive a reward.

In contrast to temporal measurement, temporal production signals corresponding with the passage of time can be generated completely internally and need not have explicit environmental correlates. To investigate such a completely internal timing mechanism, we designed a task that requires animals to move consistently at regular time intervals without any external or environmental cues (Figure 1A,B). Specifically, the task requires the animals to make rapid eye movements (saccades) back and forth between two fixed targets every second. Trials were immediately aborted if any intersaccadic interval, the time between subsequent saccades, differed by more than 200 ms from the 1 s standard. The lack of any external timing-related visual cues serves to control for sensory anticipation and temporal measurement. Trial length and reward amount were randomized on a trial-by-trial basis to minimize reward anticipation. We further dissociated reward from the saccadic movement by allowing trials to end at any time within an interval, not just immediately following the completion of a saccade. Finally, by utilizing saccades instead of other movements (such as reaching), we minimized any variability in motor output since saccade metrics between two fixed locations are highly consistent.

After animals were trained to consistently saccade at 1 s intervals, we recorded from individual neurons in the lateral intraparietal area (LIP). Our recordings confirm previous suggestions of temporal representations within the area but suggest that the nature of these representations is far different from prior reports. First, we found that unlike the activity observed in previous studies, activity within LIP was characterized by a constant decrease in activity throughout the timed interval prior to movement initiation. Second, activity throughout intersaccadic intervals was significantly predictive of interval duration on an interval-by-interval basis. Lastly, the sign of the correlation between activity and behavior depended on saccade direction. Therefore, it appears that LIP activity contributes to both saccade initiation and fixation, and temporal production in our task reflects the balance between these two signals.

## Results

We trained two monkeys (*Macaca mulatta*) to perform a variant of the delayed-saccade task [36],[37] called the self-timed rhythmic-saccade task (Figure 1A,B). The task was designed to focus on temporal motor production and avoid any regular pattern of sensory stimulation or reward that might lead to temporal measurements within the task. In this paradigm, the monkey was required to rhythmically saccade back and forth between two static targets (red and blue) at a defined interval so that saccades occurred each second. Because there were no external cues regarding this interval, the monkey had to form and follow an internal and explicit temporal representation to successfully perform the task [23]. Saccades made toward the neuron's response field (RF) are referred to as “peripheral” saccades (blue to red), while saccades made away from the RF (or towards the central target) are referred to as “central” saccades (red to blue).

**Figure 2:**
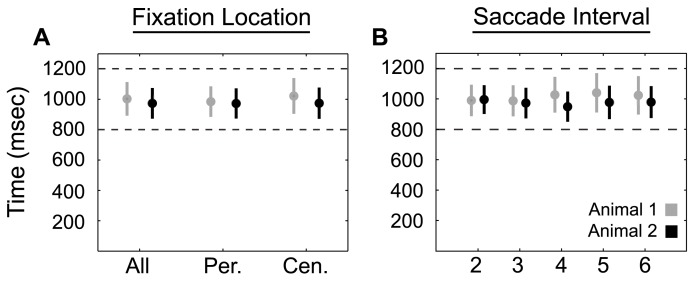
Intersaccade durations by direction (A) and sequence number (B) for both animals. (A) Average intersaccade durations (dots) by direction of movement with standard deviations (bars). Intersaccadic intervals were calculated prior to the upcoming saccade indicated on the *x*-axis. (B) Same as in (A) showing intersaccade durations by sequence number for the first five saccadic intervals that displayed static targets. Intersaccadic intervals were tightly distributed around the trained interval of 1,000 ms with standard deviations less than the allowable error (±200 ms, dashed lines in A and B). Interval durations are similar between directions and intervals within each animal and between animals. Gray color indicates animal 1. Black color indicates animal 2.

To verify that the animals learned the trained interval of 1 s, we examined the produced intersaccadic intervals as a function of saccade direction and serial order during the rhythmic task (Figure 2). A total of 78,059 saccades (average of 781 saccades/cell, with a standard deviation of 235 saccades/cell) were analyzed over 19,177 trials. We found that both animals displayed highly consistent behavior, with standard deviations much smaller than the allowable behavior window of ±200 ms. Interval production depended neither on saccade direction nor serial order. Animal 1 produced an average intersaccadic intervals of 1,003 ms (standard deviation of 111 ms), 1,022 (118), and 985 (101) prior to all, peripheral, and central saccadic movements, respectively. Animal 2 produced those same average intersaccadic intervals at 973 ms (101 ms), 974 (103), and 972 (100), respectively. Average intersaccade times for the first five intervals serially (excluding the first) for Animal 1 are 991 ms (104 ms), 988 (102), 1,028 (118), 1,041 (129), and 1,024 (126). Animal 2 produced average intervals of 984 ms (94 ms), 971 (101), 948 (99), 976 (110), and 978 (105). Because the behavioral comparisons within each animal and between animals are very similar, all future analyses will combine data for all saccadic intervals (except first intervals) and both animals.

We also compared successive intersaccadic intervals produced by the animals during the self-timed rhythmic-saccade task. Previous studies have noted that a negative correlation exists between subsequent repetitive behaviors, such as finger taps or saccades [38]–[40]. However, these studies focused on tempo reproduction rather than a self-timed behavior. An investigation of successive intersaccade times in our self-timed task revealed that the combined behavioral data displayed a small but significantly positive correlation (*r* = 0.05, *p*<0.0001). The lack of *r* value consistency with previous studies is likely due to task differences. Unlike tempo reproduction, our task resets the required timed interval after each movement. Thus, there is no behavioral advantage to compensating for a short interval with a long one, as in a tempo reproduction task.

**Figure 3:**
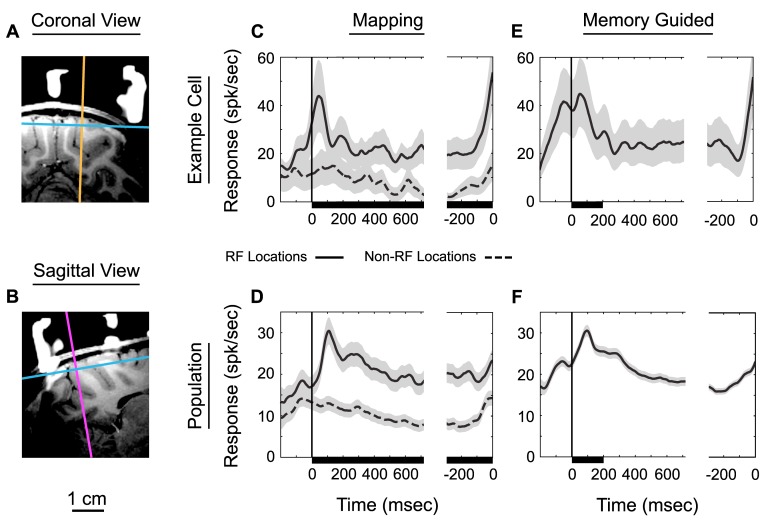
MRI/CT co-registered images of a recording penetration (A and B) along with average neuronal responses of an example cell and the population during mapping (C, D) and memory-guided delayed-saccade tasks (E, F). The orange line in the coronal view (A) represents the medial/lateral axis at which the chamber was oriented, while the magenta line in the sagittal view (B) represents the anterior/posterior axis. Those lines also represent an electrode path through LIP for which 16 of the neurons from animal 2 were recorded over a span of 5 mm. The blue line in each image represents the transverse plane of the chamber and also indicates the superior limit for which neurons were recorded. (C) Average response of an example cell during RF mapping. The solid line corresponds to targets within the RF, while the dashed line corresponds to targets outside of the RF. (D) Same as (C) except for population mapping responses combining animals 1 and 2 (*N* = 39 cells). (E) Average memory-guided delayed-saccade activity for the same example cell shown in (C). (F) Average population response for animals 1 and 2 combined for the same tasks in (E) (*N* = 100 cells). Plots are aligned to two events in the trial: left, target onset; right, saccade onset. Black bar along *x*-axis represents the time that the peripheral “non-fix” target is displayed during mapping and memory-guided trials. Gray shading indicates standard deviation of the mean.

In order to investigate the neural basis of this temporally precise behavior, we recorded individual neurons from a parietal area that has previously been implicated in timing, the lateral intraparietal area (LIP; Figure 3A,B). We first examined the activity of individual neurons during a mapping task (Figure 1C) to ensure that the neurons we recorded displayed spatially selective activity typical of LIP neurons. Consistent with previous reports, we found spatially specific stimulus onset responses and presaccadic buildups in activity (Figure 3C,D).

Many neurons within LIP also exhibit stereotyped delay period activity during tasks in which the location of a transiently presented saccade target must be remembered, such as the memory-guided delayed saccade task [41]–[43]. As seen for both an example neuron (Figure 3E) and our population (*N* = 100, Figure 3F), the flashed target within the RF (black bar) elicited a transient increase in activity followed by sustained activity during the delay period (the time between the RF target being extinguished and the fixation point being extinguished). This sustained activity remains above activity levels that were observed during mapping trials for non-RF locations (dashed lines in 3C,D). Additionally, many neurons in our population displayed pre-saccade-related activity (59/100 showed this trend, while in 25/100 this increase was significant; *t* test, *p*<0.05). This steady increase in activity is evident before saccade onset and can be seen in both the example cell and population activity. Such pre-saccadic activity has previously been attributed to saccade planning [34],[41]–[43]. However, this increase could also be due to reward expectations or changes in attention closely linked to the required movement [24]–[29].

To quantify the level of delay period activity [34], we calculated a memory index (MI) by dividing the average delay activity (320–720 ms following target onset) by the average visual activity (0–320 ms following target onset) [16],[44]. Although on average our MI was larger than some reported previously (mean = 0.78, SD = 0.26 versus mean = 0.52, SD = 0.63 [44]), this difference is not significant and to be expected given our exclusion of cells with no delay period activity. Also consistent with previous reports, we found a significant correlation between the MI and presaccadic activity (*r* = 0.22, *p*<0.05 versus *r* = 0.30, *p*<0.05; [44]).

After this memory-guided task, we recorded from the same neurons while the animals performed the self-timed rhythmic-saccade task (Figure 1A,B). In the following analyses concerning the self-timed rhythmic task, the first interval was excluded because of the predictable onset of the subsequent target, which distinguishes the first interval from all subsequent intervals. As was done with the behavior, firing activity was segregated based on direction and the interval number within a trial (Figure S1). The firing rate for each direction and interval (first intervals done separately) was then analyzed in order to determine if the activity varied from interval to interval within a trial. No significant correlation was found between neuronal activity and interval number for either monkey (*p*>0.05). Therefore, as with the behavioral data, neural activity is combined across all intervals (aside from first intervals) in subsequent analyses. Because the firing activity between both monkeys was very similar, data from both animals will be combined in all future analyses as well.

**Figure 4:**
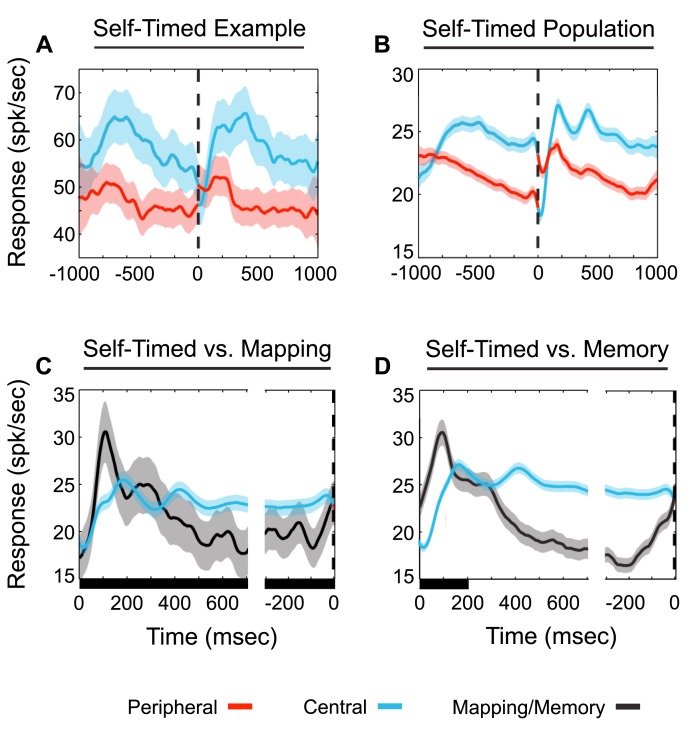
Neural responses during self-timed, mapping, and memory saccade tasks. (A) Average neuronal response of an example cell (*N* = 1 cell). (B) Average combined population activity during the self-timed task (*N* = 100 cells). Zero time point (vertical dashed line) indicates time of saccade onset. Red lines indicate response periods during peripheral target fixation, while blue lines indicate response periods during central target fixation. All traces in (A and B) are aligned to saccade onset. Activity decreases at a constant rate prior to both directions of saccades. (C) Average self-timed (*N* = 100) and mapping responses for the subset of our data with complete mapping runs (*N* = 39). Black line indicates response obtained during the mapping task (aligned to peripheral target onset on left and peripheral saccade onset on right), as in Figure 3C. The blue line indicates periods of central fixation (blue shading) aligned following central saccade onset (left, C) and prior to peripheral saccade onset (right, C). (D) Same as in (C) except the comparison is between self-timed (blue) and memory trials (black, Figure 3D) (*N* = 100). Shading along the *x*-axis in (C and D) represents the presence of a target stimulus within the RF. Activity during intersaccadic intervals of the self-timed task is different from what is observed from the same cells during mapping and memory-guided saccade tasks in which only a single saccade is required, the reward can be anticipated, and the saccade is cued with a stimulus event (C and D). Shading of firing activity represents standard deviation of the mean.

Figure 4A shows the neural activity of an example cell as the monkey performed the self-timed saccade task. The dashed vertical line represents saccade initiation. Blue to red traces are aligned to peripheral saccades (saccades to the peripheral or RF target), while red to blue lines are aligned to central saccades (saccades to the central target). The combined, average population activity for all 100 neurons is shown in Figure 4B. As expected, irrespective of the interval, response rates are consistently higher during the central fixation when a target is within the RF (blue) than during peripheral fixation (red) when there is no target within the RF. However, in contrast to expectations from memory-guided tasks, there is no significant pre-saccadic buildup in activity for the example cell or the population. As reflected in the population activity, the majority of the neurons (81/100) did not display significant increases in activity immediately prior to saccades. On the contrary, as evidenced by the population averages, activity decreases prior to saccade initiation for both directions of movement (Figure 4B).

To isolate the factors that could cause temporal modulations of activity prior to saccades, we compared the activity observed in the self-timed saccade task with activity observed in mapping and memory-guided saccade tasks for the same fixation location (central fixation) (Figure 4C,D). The left portion of the plots are aligned following a central saccade (blue) or target onset (black), while the right portion of the plots are aligned to peripheral saccade onset (blue and black). In all of these tasks, the actual upcoming movement (a peripheral saccade) and the timing (a 1-s interval) are consistent. However, the stimulus events and rewards associated with this planned movement are different. For mapping trials, although the peripheral target remains lit for the entire interval, a visual cue tells the animal when to move (central fixation point turns off) and the animal only makes one saccade prior to receiving a reward. For memory trials, the peripheral target is only lit for the first 200 ms of the trial, the animal is cued when to move, and the animal only makes one saccade prior to reward delivery.

In the mapping task, a transient increase in activity occurs following RF stimulus onset. A similar response is also evident during the self-timed saccade activity (blue, left), although the cause is likely due to a central saccade bringing the RF to encompass the peripheral target instead of target onset since first saccades are not included in the rhythmic analysis (Figure 4C). After the initial response, firing rates decrease in both the mapping and self-timed tasks. However, during this period the mean firing rate is consistently higher in the self-timed task, in which the animal cannot rely upon an external cue, than in the mapping task, in which such cue is available. This difference in firing rate is therefore consistent with a temporal production signal.

Similar differences are observed when the responses prior to memory-guided and self-timed saccades are compared (Figure 4D). Notably, the mapping response is virtually identical to the memory-guided response, indicating that the presence or absence of a stimulus within the RF prior to the saccade cue has a minimal effect on responses. This is also consistent with a temporal production signal, rather than any sensory effects, dominating LIP responses during the self-timed task. Immediately prior to the saccade, activity rises much more in both the mapping and memory guided tasks (black) when the animal knows that a reward is imminent than in the self-timed task (blue). This difference suggests that the pre-saccadic rises in activity reported in previous studies may reflect specifics of the task (such as reward or sensory anticipation), rather than generalized patterns underlying saccadic timing.

Although the predominant feature of activity modulation during self-timed saccades is a near linear decline in firing rate over time, other modulations are clearly present. Around the time of saccade onset (±100 ms), the activity displays distinct modulations. Brief increases in activity just prior to saccade onset are followed by short intervals of decreased activity at the time of saccades. These peri-saccadic modulations in activity are similar between saccade directions and are consistent with previous studies as being signals of a global remapping of the RF [45]–[48].

The largest deviation from the overall decline in activity is the sudden increase in activity immediately following central saccades (blue, 0 to 250 ms) (see Figure 4B). The increase in activity immediately following a central saccade is consistent with bottom-up sensory stimulation because, as an immediate consequence of the central saccade, the peripheral target is moved into the neuron's RF. A large increase in activity is also visible early within the interval prior to peripheral saccades (red, −1,000 to −700). Since saccades are performed back and forth between the two targets and since all trials outside of the first intervals are analyzed together, this increase may also represent sensory stimulation of the RF.

**Figure 5:**
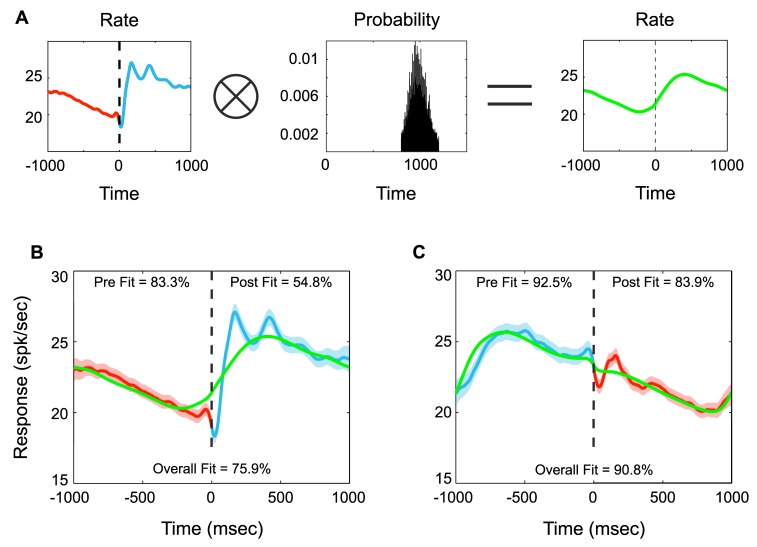
Neuronal activity predictions on the assumption that only one type of saccade modulates activity. (A) Example calculation of predicted activity. The actual firing rate aligned to central saccades (left, red to blue) is convolved with the intersaccade distribution times of peripheral saccades (middle, black) in order to produce the predicted rate (right, green). (B) Actual averaged neuronal activity (red to blue) and predicted activity (green) with error measurements (% fit) between actual and predicted activity, aligned to central saccades (moving away from the RF). (C) Same as in (B) except activity (blue to red) and prediction (green) are aligned to peripheral saccades (moving toward the RF). Shading of firing activity shows standard deviation of actual firing rates. Datasets (B and C) differ only in their saccade alignment.

The cyclical nature of the task makes it difficult to ascribe firing rate changes to particular saccades because, for example, firing rate changes following central saccades are also preceding peripheral saccades. To address this ambiguity, we took advantage of the variability in intersaccadic intervals. Specifically, we looked at whether activity locked to a particular saccade could completely explain the peri-saccadic activity aligned to the other saccade by generating firing rate predictions of each saccadic alignment on the basis of the other (Figure 5A) and behavioral variability. A good fit between the predicted rates (green traces) and the observed firing rates (red and blue traces) would indicate that activity locked to a particular saccade can largely explain the firing rate changes seen in the cyclical task. Conversely, a poor fit would suggest that activity aligned to a particular saccade cannot solely account for the observed neural activity. For example, if LIP activity were strongly modulated by both central and peripheral saccades, then a firing rate reconstruction based on only one of those saccades would poorly predict peri-saccadic activity for the other saccade. However, if the fit was good for one type of saccade and relatively poor for the other, it would indicate that firing rate modulations could be largely explained by only one of the two saccades.

In general, the predicted rates were similar to the actual firing rates, consistent with an overall decrease in firing rate being the dominant activity modulation during intersaccade intervals (Figure 5B,C). However, there are a few instances for which the predicted activity poorly fits the observed activity. The first of these is the time period just before and just after both directions of saccade initiation (±100 ms), which, as mentioned previously, is consistent with previous reports of RF remapping signals in LIP [46]. The second significant discrepancy is during the 250 ms immediately following central saccades (Figure 5B). By contrast, peripheral saccade aligned activity (Figure 5C) during the corresponding period of time (−1,000 to −700 ms) is well fit. This suggests that the sudden increase in activity immediately following central saccades, which is likely explained by the saccade-related movement of the stationary peripheral target into the RF, is largely sufficient to explain the gradual increase seen approximately 1 s prior to peripheral saccades. Moreover, activity aligned to central saccades (green, Figure 5C) is better at predicting the firing rate variations seen in the rhythmic task than activity aligned to peripheral saccades (green, Figure 5D).

Thus, the activity modulations seen in our task can be largely explained by a gradual decrease in activity following the appearance of the peripheral target in the RF. The consistency and strength of responses immediately following central saccades suggests that it could serve as a “reset” signal for timing. The linear rate of activity decrease that follows this reset could then be used to accurately measure the passage of time. However, although suggestive, these modulations need not have any relationship to timed behavior. For example, although activity decays at a constant rate following the introduction of a stimulus into the RF, this decay might not have anything to do with how the animals actually timed their behaviors and may simply reflect some intrinsic decay constant. In such a situation, activity fluctuations in LIP that are due to noise or some uncontrolled variable such as attention would have no correspondence with fluctuations in the timed behavior.

**Figure 6:**
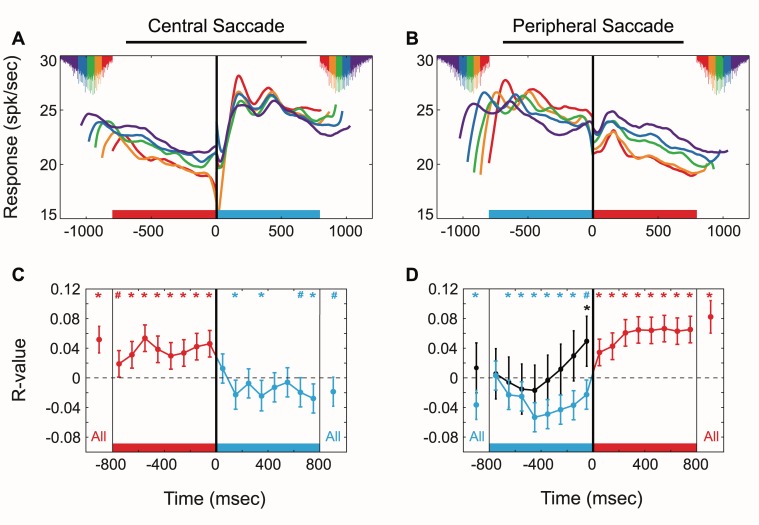
Perisaccadic firing activity segregated by interval length (A, B) and corresponding saccade by saccade correlations between firing rate and interval length (C, D). (A and B) Average combined population activity, grouped by interval length, aligned to central saccades (A) and peripheral saccades (B). Zero time points and vertical black lines indicate saccade onset. Intersaccade distribution times are shown at the beginning and end of each interval and the coloring corresponds to the firing rates. Blue bars along the *x*-axis represent time intervals during which animals were fixating on the central target (peripheral target located in RF). Red bars represent time intervals during which animals were fixating on the peripheral target (no stimuli in RF). Central Fixation Intervals: red, 0.08–0.89 ms; orange, 0.90–0.94; green, 0.95–0.99; blue, 1.00–1.05; purple, 1.06–1.20. Peripheral Fixation Intervals: red, 0.80–0.91 ms; orange, 0.92–0.96; green = 0.97–1.01; blue, 1.02–1.07; purple, 1.08–1.20. (C and D) Binned correlations between firing rate and current interval lengths for all intervals of the combined population, aligned to central saccades (C) and peripheral saccades (D). First and last bins (C, D) display correlation of the 800 ms of the interval (“All”, −800 to 0 ms, or 0 to 800 ms) about saccade onset. Black points in (D) correspond to correlations observed during the memory-guided saccade task. Horizontal colored bars along the *x*-axis represent the 800 ms of the intervals over which correlations were made. The 800 ms intervals were investigated as that is the minimum time for correctly made saccades as defined by the error window (1,000 ms±200 ms). Asterisks and number signs indicate bins that are significantly correlated (* *p*<0.005, # *p*<0.05). For central saccades (C), the *p* values from left to right are: 5.5e-13; 0.0164, 0.0001, 0, 0, 0.0001, 0.0001, 0, 0, 0.0502, 0, 0.497, 0.0012, 0.0827, 0.958, 0.0126, 0.0004; 0.0163. For peripheral saccades (D), the *p* values from left to right are: 5.5e-7; 0.734, 0.0049, 0.0046, 0, 0, 0, 0.0001, 0.0174, 0.0001, 0, 0, 0, 0, 0, 0, 0; 8.49e-28. Bars represent the 99% confidence interval for each bin. The horizontal line represents an *R* value of zero for reference. Time bins are correlated to the current intervals represented by the colored bars along the *x*-axis. Because of different selection criteria for t<0 and t>0, slight discontinuities in firing rate are visible at t = 0.

To examine this possibility, we studied whether LIP activity fluctuations during intersaccadic intervals were predictive of the animals' actual saccadic interval. Figure 6A,B show the firing activity as a function of interval length for each saccade direction. Each trace is an average rate of one-fifth of the trials, and interval lengths are sorted based on current intervals both prior and subsequent to the saccade displayed at time point 0 (activity prior to the saccade is sorted based on the interval that ends at 0 and activity following the saccade is sorted based on the interval that begins at 0). The red traces represent the shortest fifth of intervals produced by the animals, while the purple traces represent the longest fifth of intervals (as indicated by the intersaccade distribution times shown at the beginning and end of each interval). The red and blue bars at the bottom of each figure indicate fixation location during each interval (red, fixation of peripheral target; blue, fixation of central target). The consistent ordering of firing rates with respect to interval length suggests that activity is predictive of the behavioral interval.

To further examine the relationship between activity and behavior, we computed the correlation between neural activity and the intersaccadic period. We investigated this relationship over the 800 ms before and after saccade initiation by looking at correlations over 100 ms bins (Figure 6C,D). Correlations were calculated on an interval-by-interval basis across all trials of all cells. For each bin, the action potentials were summed and analyzed with respect to interval length. Correlations prior to saccades were analyzed using intervals that ended at time 0, while correlations following saccades were analyzed using intervals that began at time 0. In this way, all correlations are concerned with current intervals associated with upcoming saccades. This analysis allows us to examine whether time-related signals are consistently present throughout intersaccadic intervals or more prevalent immediately before or after saccades.

Activity at the peripheral location was consistently predictive of interval duration irrespective of alignment (red, Figure 6C,D, correlation analysis, * *p*<0.005, # *p*<0.05). These correlations are notable in two respects. First, they occurred during a period of time when there is no sensory stimulation in the RF and the RF is not a potential target. Second, and consistent with the orderly segregation of the traces in Figure 6A,B (red shaded intervals), the correlations were positive, meaning that increases in activity were associated with increases in the intersaccadic interval. This is the opposite relationship to what would be expected by previously proposed activity-based threshold models of timing [4],[22] and sensory integration [49], in which increases in activity are associated with reaching a threshold earlier.

Activity at the central location aligned to peripheral saccades also displayed significant correlations to interval length throughout nearly the entire intersaccade period (blue, Figure 6D, * *p*<0.005, # *p*<0.05). However, in this case, the correlations are primarily negative. Surprisingly, the activity aligned to follow central saccades corresponding to the same fixation location does not consistently show significant correlations throughout the interval (blue, Figure 6D, *p*>0.05). This difference suggests that certain events, such as the sensory-driven response transient following central saccades which dominates response modulations in our task (Figure 5), can mask temporal production signals.

Correlations between firing rate and saccade metrics (saccade velocity) were also calculated in order to determine if LIP activity was related to saccadic motor output. We found that the overall population activity was not significantly correlated to saccadic velocity (*p*>0.05). Therefore, LIP activity is likely related to motor planning rather than saccade metrics. Further support for motor planning is provided by the difference in the correlations between the self-timed task (blue) and the memory task (black) (Figure 6D). Although these tasks are similar in that the same movement is required after the same delay, significant correlations only exist throughout the interval for the self-timed task.

Since firing rates throughout the delay periods of these intervals are largely predictive of the interval length, this suggests that activity within LIP is a temporal production signal that can be utilized in order to time saccade initiation. Consistent with previous reports we found that a subset of individual cells were correlated to interval duration (10/100 prior to central, 8/100 following central, 11/100 prior to peripheral, 3/100 following peripheral, *p*<0.05) [22]. The sign of the cells' significant correlations typically had the same sign as population correlations. The low number of neurons displaying significant correlations between rate and interval length suggests that the timing signals of LIP in our task are most prominent at the population level but too weak to be observed in the activity of most individual neurons over the time period of our recording sessions.

When the activity of each individual neuron was normalized (*z* score) prior to calculating the correlations, the correlation values throughout the interval generally maintained the same sign (unpublished data). However, the *r* values were consistently dampened. This suggests that neurons with greater modulations in activity contribute more to behavioral timing. When we looked at neurons with higher degrees of rate changes, we found that activity displayed stronger correlations while the animals were fixated at the peripheral target (Figure 6, red bars). Overall correlations were significant for precentral and postperipheral saccade aligned activity and increased from 0.050 and 0.076 to 0.145 and 0.157, respectively. Yet correlations were not significant for either saccade alignment as animals fixated on the central target in the high modulation cells. Cells with lower modulations in activity still displayed significantly correlated activity both prior to (*r* = −0.042) and following (*r* = 0.039) peripheral saccades, although the correlations following peripheral saccades were reduced compared to the combined population. These differences between cells with high and low modulations may support the idea that there are two populations of neurons within LIP [22], each of which contributes to timing differently. However, since both populations of cells do contribute to the timing of saccades, we will continue to discuss temporal production for the entire combined population.

Although we observed that LIP activity is significantly predictive of current interval length, other studies have shown that parietal activity can also represent past and future events [29],[50]. To determine if LIP activity is also related to past and future intervals in our task, we performed a regression analysis where we examined the relationship between neural activity with past, current, and future interval lengths. Because fixation location and the presence of a target within the RF strongly modulates responses, this analysis was done separately for both locations (central and peripheral). Firing rates for this analysis were obtained from the 800 ms adjacent to saccade onset for the intervals with the highest overall correlation values. We found the population activity to be significantly related to the current intervals at both locations (linear regression coefficients: at center = −9.4 spikes/s/s, at RF = 7.9 spikes/s/s, *p*<0.005), but not with past or future intervals (*p*>0.05). Very few individual cells were significantly related to any intervals. Only 0, 1, and 0 cells were significant for past, current, and future intervals, respectively, while the animals were fixated at the central target. Similarly, no cells were significant while fixated at the peripheral target (regression analysis, *p*>0.05). Therefore, it appears that neither future interval planning nor past interval production significantly contributed to LIP activity, and the relationship with current intervals is due to population activity.

The change in the sign of the correlation between activity and timed saccades suggests that a push-pull mechanism may underlie temporal production in our task. Activity prior to a RF (peripheral) saccade “pushes” for saccade initiation in that more activity during this time leads to a faster onset of the behavior (negative correlation). By contrast, activity prior to a central saccade “pulls” for maintaining the peripheral position. In our task, the hemisphere containing the response fields corresponding to the impending saccadic target would be “pushing” for a saccade, while the opposite hemisphere would be “pulling” or delaying a saccade. Because activity fluctuations are associated with the same change in timing no matter when they occur within the intersaccadic interval, the mechanism must involve the integration of activity with very little decay [49]. However, in contrast to previous proposals, LIP activity cannot be a direct proxy of a saccadic decision variable, since activity neither rises over time nor is it associated with a constant value immediately prior to saccade onset (Figure 4).

**Figure 7:**
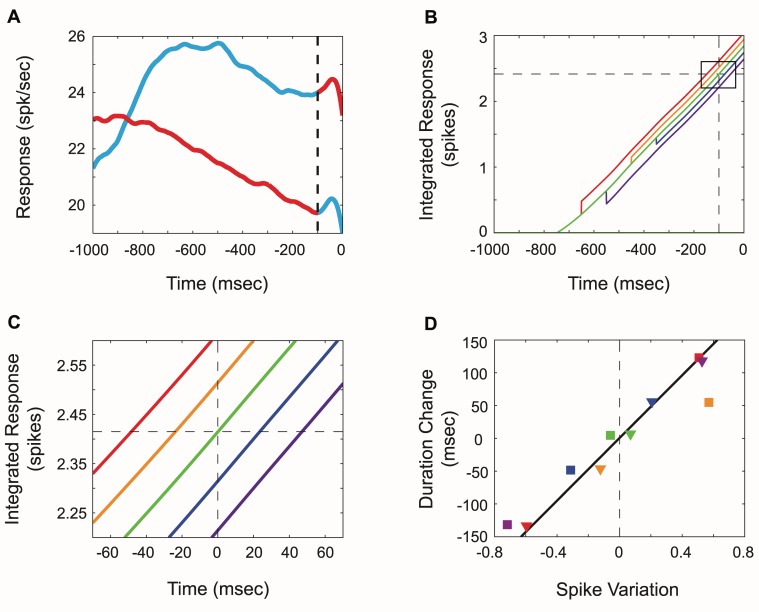
Difference model of cross-hemispheric LIP activity driving temporal production. (A) Actual average LIP population activity aligned to saccades made to the peripheral target (blue to red) and the central target (red to blue) (same as in Figure 4B, −1,000 to 0). The vertical dashed line represents the time at which saccades are signaled (motor delay accounts for the remaining 100 ms prior to saccade initiation). (B) The green line displays the difference between the integrated rate aligned to the peripheral target (A, blue to red) minus the integrated rate aligned to the peripheral target (A, red to blue) prior to saccade initiation (only the positive value differences are shown). The colored lines show how the intersaccadic interval can be altered based on changes to the integrated difference between the rates shown in (A). Altered spike counts: red, +0.2 spikes; orange, +0.1; blue, −0.1; purple, −0.2. Because of perfect integration, changes in activity at any point during the interval can alter saccade timing. The horizontal dashed line represents the threshold, while the vertical dashed line represents the time at which the green line crosses this threshold. (C) A zoomed view of (B) (black box inset in B) illustrating the behavioral timing predictions of the model (matched color scheme from segregated firing rates in Figure 6A,B). (D) As shown in (B and C), the model predicts a linear relationship between activity variations and variations in the intersaccadic interval (black line). Actual observations of integrated activity variations when segregated according to intersaccadic interval are plotted for central (squares, Figure 6A) and peripheral saccades (triangles, Figure 6B). Activity variations were computed by comparing the activity for a particular interval range (Figure 6) with the average activity across all intervals (A). Consistent with the differencing model, positive differences in activity (when compared to the average) prior to central saccades (e.g., purple square) are associated with decreases in the interhemispheric activity metric used to predict behavior (negative spike variation in D). The data demonstrate that activity variations prior to both central and peripheral saccades are consistent with the linear difference model.

The simplest realistic model is therefore one in which a difference between the temporally integrated activity of the two LIP locations representing the targets underlies the decision to saccade. Because we have data for saccades both towards and away from the RF, we can use the average population data of Figure 4 to construct such a model. Specifically, we integrate the activity over the cells representing the impending target and cells representing the “anti-target.” When such integrals are differenced, the result is a signal that increases in a near linear fashion throughout the interval prior to saccade initiation (Figure 7B,C, black trace). A threshold is then applied (Figure 7B,C, dashed horizontal line) so that when the “push” of the RF location outweighs the “pull” from an opposite location by a set amount (threshold), a saccade is signaled. On average, this threshold is reached at the beginning of the presumptive peri-saccadic remapping signal, consistent with typical latencies between LIP activity and saccades (∼100 ms) [51].

In the absence of any activity fluctuations from the responses shown in Figure 7A, this model would produce completely regular intersaccadic intervals. However, in the presence of activity fluctuations in one hemisphere, either due to noise or changes in an uncontrolled variable such as attention, this regularity changes, allowing for a variety of intersaccadic durations. Because of the differencing operation, fluctuations at the different locations have opposite effects (Figure 7B,C). Because of the integration, brief activity fluctuations anytime during the interval have an equivalent effect on intersaccadic duration. Thus, a momentary increase (or decrease) in activity will have an identical effect of the timing of the impending saccade no matter when it occurs. To test how this model compares with experimental observations, we plotted how differences in the integrated activity would increase or decrease the intersaccadic interval with the same constant threshold (Figure 7D, black line) and compared these predictions with the activity differences observed in our interval sorted response plots (Figure 6A,B). The model (slope = 239 ms/spike, black) accurately predicts the average responses prior to central (Figure 6A, squares in Figure 7D) and peripheral (Figure 6B, triangles in Figure 7D) saccades for timed intervals of different durations.

## Discussion

In order to investigate neural activity related to temporal production, we devised a self-timed rhythmic-saccade task that controlled for temporal measurements while minimizing sensory and reward anticipation. Animals were required to make saccades back and forth between two fixed targets at a fixed interval so that saccades occurred each second (Figure 1A,B). We found a systematic decrease, rather than an increase, in activity within LIP prior to saccades. The systematic decrease in activity was significantly predictive of intersaccadic interval length (correlation analysis, Figure 6). The relation to interval length was found to only be significant for current (not past or future) intervals (regression analysis), but the sign of the correlation between activity and timed intervals depended on the direction of the impending saccade.

The animals in our study displayed the ability to precisely and consistently produce a rhythmic behavior very near the trained interval (Figure 2). This was true regardless of the direction of the saccade (peripheral or central) or the saccade number (second, third, etc.).

However, aspects of our results are not consistent with previous studies that investigated motor timing in repetitive behaviors. One timing model used to describe rhythmic saccades [40] was developed by Wing and Kristofferson from studies utilizing finger tapping [38],[39]. The model stipulates a negative correlation between subsequent timed repetitive movements. For example, if a saccadic interval is longer than the trained interval, then the following saccadic interval is likely to be shorter. This negative correlation helps ensure accuracy in tempo replications, since short intervals can be compensated by longer intervals (and vice versa) in order to stay on beat. This negative relationship has been attributed to the variability of motor output delay and the idea that chance variations about the mean delay will tend to produce a negative correlation between adjacent intervals [52].

In our design, we failed to find a negative correlation between subsequent timed intervals. Instead we found a small (*r* value = 0.05), but significantly positive correlation (*p*<0.0001) between subsequent saccadic intervals. The fact that our results do not display the negative correlation described by the Wing and Kristofferson model likely reflect task differences. Unlike our task, these other studies did not require their subjects to precisely execute a trained interval. Instead, the subjects first followed along with a cued motor sequence and then continued the motor task in a self-paced manner after cue extinction. There were no repercussions for imprecise timing as in our task (trial ends, no reward). Additionally, our task resets the trained interval following each saccade. This means that the better the animal is at precisely producing the trained interval on each saccade, the better chance it has of receiving a reward. In these other studies, the subjects attempted to replicate a tempo that is not reset with their behavior. Therefore, the Wing and Kristofferson model may only be useful in describing tempo replication and may not reflect general mechanisms governing temporal production.

The near independence between adjacent timed intervals suggests that the underlying temporal production signal is being reset by each saccade. The notion of saccades effectively resetting time keeping in our rhythmic task is consistent with our physiological observations in several respects. First, as evidenced by saccade aligned firing rates, a similar up-down peri-saccadic modulation in firing is present irrespective of saccade direction. These modulations in activity occur approximately 100 ms prior to saccade initiation, similar to saccadic latency times within LIP [51]. Therefore, remapping may serve as a reset signal. Second, as would be expected by a reset, activity in LIP was only correlated with the current behavioral interval within a sequence and not with past or future temporal production. Third, the relationship between activity and behavior flips after a peripheral saccade from negative to positive.

Neurons within our sample exhibited response properties largely consistent with previous reports from LIP. They had spatially specific response fields depending on the direction of the saccade target (Figure 3C,D), responded to target onsets within those response fields, maintained responses when delays were imposed between target extinction and saccade initiation (Figure 3E,F), and showed peri-saccadic modulations (Figure 4) consistent with remapping [46]. Our population of neurons also displayed task-dependent anticipatory behavior. For instance, increases in activity are observed just prior to saccade onset for tasks in which the movement is cued and only a single saccade is required (Figure 3C–F). However, this same level of presaccadic increase in activity is not apparent in the population activity during the self-timed task (Figure 4A,B). This suggests that climbing activity may be associated with sensory and/or reward anticipation rather than motor anticipation.

Response rates were consistently higher prior to saccades in the self-timed task than saccades in the memory-guided task. If there were saturation issues, this higher response level might preclude our ability to observe presaccadic buildups. We consider this unlikely for two reasons. First, the mere presence of an RF stimulus does not preclude our ability to see presaccadic buildup, since a buildup is evident during the mapping task (Figure 3C,D). Second, the light-sensitive response early within the interval (Figure 4A,B) shows that the population is capable of higher rates of firing.

In our self-timed task, although activity consistently declined over time, the mean levels of activity were significantly different for the two fixation locations (Figure 4). Because the “real-world” or head-centered positions of our targets remained constant throughout the trials (Figure 1A,B), this response difference could be due to the presence or absence of a target within a retinotopic RF. However, as can be seen in the comparison of mapping to memory-guided responses, the presence or absence of a stimulus within the RF during a 1 s interval had very little effect on responses (Figure 3D,F). In both cases, a low-latency stimulus-evoked onset response is followed by a gradual decrease in response during the delay period and then an increase in activity prior to the saccade, and the response rates during all of these phases are virtually identical.

Another potential complication is that eye position varies between the two intersaccadic intervals. Approximately 50% of LIP neurons are modulated by eye position [41]. In many cases, this modulation can be described as a gain effect, in that visual and delay responses are consistently modulated by a single factor according to eye position. For example, in one study, 30% variations in response magnitudes were reported over eye-position ranges of 45 by 45 deg [41]. Although this is approximately the modulation seen between our central and peripheral locations (Figure 4B), it is unlikely that an effect that is only observed in half of LIP neurons would give this degree of modulation over the eye-position shifts (typically around 15 deg) in the self-timed task. Furthermore, because the direction of the eye position effects is not consistent between neurons, any effect seen in individual neurons would average out to zero over a relatively large sample such as ours. Finally, as a part of our eye calibration procedure, the position of the central fixation point on the screen varied from trial to trial along the corners of a 4 deg square, which would further reduce any eye position effects.

Thus, we feel that the most likely explanation for the difference between central and peripheral fixation is the well-established sensitivity of LIP responses to the location of the impending saccade. However, even if other factors contribute to this response difference, the interpretation of our data with regard to timing remains unaltered. Because during a single intersaccadic interval neither the stimulus nor the eye position is changing, response changes during this interval cannot reflect these factors. Since all visual stimuli are stationary and the reward cannot be anticipated, the only factor that consistently varies over the intersaccadic intervals is the passage of time. Consistent with a role in the representation of time, we found significant correlations on an interval-by-interval basis between neuronal activity in LIP and the duration of this interval. Because these correlations are present for both locations, they are robust to changes in eye position, direction of the impending saccade, or whether there is a stimulus within the RF. Since an internal sense of time is the only cue available to the monkey with which to initiate saccades, we have interpreted this correlation, which, to our knowledge, has never been previously reported, as reflective of a temporal production signal.

The specificity of these correlations, which are absent in a task not requiring timing (Figure 6D, black) and reverse sign according to the specific saccade which is impending (Figure 6C,D, blue and red), rule out that they are the result of a generalized vigilance or task-related factor.

However, it is also possible that LIP, instead of representing information relevant to a decision to saccade, namely the passage of time, instead represents a motor plan whose execution after a decision has been made can be delayed. We consider this explanation unlikely for several reasons. First, there is no evidence to suggest that increases in activity in LIP would be associated with delays in the execution of a motor plan. On the contrary, many experiments have demonstrated that LIP activity appears to be associated with predecision information, whether that information be stimulus related [23]–[24],[49],[53]–[63] or time related [4],[16],[22],[64]. Second, we found no evidence that actual saccade metrics (e.g., velocity) depended on LIP activity. Third, because LIP activity was correlated with timing even when the RF location was not a potential target (e.g., when the monkey was fixating at the peripheral location), our results are not consistent with changes in a motor plan strictly associated with a particular retinotopic location. Fourth, if LIP activity solely reflected a motor plan, then activity fluctuations near the time of the saccade should have particularly strong correlations with behavior. By contrast, we find that behavioral correlations are relatively constant throughout the entire intersaccadic interval, even when the saccade is going to occur 800 ms in the future. Finally, since the motor plan for memory-guided and self-timed saccades are identical, one would expect little difference in firing rates or behavioral correlations, in contrast to our observations (Figures 3 and 6).

Neuronal representations of time within LIP have previously been described by climbing activity, a steady increase in neuronal activity over time to a threshold level, at which time an action ensues [4],[16],[22]. A higher rate of activity (or a faster rise to threshold) produces a shorter interval and therefore a negative correlation between rate and time. Although brief periods of increased activity can be seen in our population activity (Figure 4B), these increases can be explained by RF remapping [46] and sensory responses to the peripheral target being moved in and out of the RF as the animal produces saccades. These brief periods of increases in activity do not fit the parameters of climbing activity as a timing signal [65],[66]. Instead, the prominent pattern of activity is a steady decrease in firing rate over the delay period.

One possibility for why we observed falling, as opposed to climbing, activity prior to saccades is the differences between our task and those employed previously to study timing. Because of the close associations of sensory cues and reward that occur near the time of the behavior in previous studies, which are absent by design in our task, it is possible that climbing activity is more related to sensory and/or reward anticipation (time measurement) than motor planning. This notion is consistent with our observations of neuronal activity during a task that is much more analogous to previous studies. The same neurons that displayed falling activity during the self-timed rhythmic-saccade task displayed very different activity during the memory-guided delayed-saccade task. In the memory task, the basic behavior, namely waiting 1 s prior to making a saccade, is similar to the self-timed task. However, in terms of temporal measurement, the tasks are quite different. Specifically, in the memory task (and unlike the self-timed task), the timing of the cue to make a saccade and reward can be readily anticipated. Consistent with previous observations, a presaccadic rise in activity was observed in the memory-guided task. However, this rise is largely absent in the self-timed task, suggesting that climbing activity may reflect reward anticipation rather than a motor plan.

Given these task differences, it is also possible that distinct timing systems are responsible for tasks that require temporal measurement and those that do not. Lewis and Miall [67] have proposed that there are two distinct timing systems: an automatic system responsible for predictable intervals defined by movements and a cognitively controlled system involved in temporal measurements that direct attention. Since LIP is involved in both motor production and attentional allocation [23],[68]–[73], it may be that this area is a part of both timing systems and that the task determines which timing system is utilized. For instance, when the animal is performing an interval duration comparison task or a task for which movement is cued or immediately rewarded [4],[16], the cognitive timing system would be engaged since these tasks require the timing of discrete epochs and do not control for the attentional effects that sensory and reward anticipation can have [74],[75]. The cognitive system would employ climbing activity as its timing signal in order to time the temporal measurement-related events of the task. However, when those forms of anticipation are minimized (as in our self-timed delayed-saccade task), the automatic timing system may be engaged since the task requires saccades be made at regular intervals. This would then allow falling activity, the signal responsible for the production of the timed interval, to emerge as the temporal production signal.

If the primary role of the cognitive timing system is to direct attention, it may be particularly unlikely to play a role in our task given that the spatial positions and direction of the impending saccades are never ambiguous or subject to cognitive choice. Although spatial attention may not be required, this does not mean that attention is not allocated to the targets at some point prior to saccade initiation. However, the activity we observe during the self-timed saccade task displays a decrease in rate prior to saccade initiation, not an increase as might be associated with the increasing priority of making a saccade as time elapses [76].

Another difference between our task and previous single-saccade tasks is the potential for sequence planning in our task. Psychophysical evidence suggests that, when confronted with an array of saccade targets, subjects naturally plan entire saccade sequences [77]. The planning of entire sequences has been shown to take place in a number of brain regions and for a number of tasks [50],[78]–[83]. Additionally, a study by Seo et al. [29] showed that LIP activity contains information about past events. However, we found that neither future nor previous temporal production significantly contributed to the activity of our population during this task (regression analysis, *p*>0.05). Also, our observation of near independence between adjacent intervals (correlation value of 0.05) is not consistent with sequence planning. These data suggest that, presumably because we reset the behavioral requirement after each saccade, both the animals and our neural population are concerned solely with timing single intervals within the rhythmic sequence.

The observation that activity is only correlated with the present interval implies that the correlations observed prior to central saccades to timing do not reflect past or future planning of peripheral saccades. This correlation, which gave rise to our “push-pull” model, is inconsistent with the belief that LIP RFs are exclusively tuned for contralateral visuomovement space [84]. In a purely retinotopic framework, activity prior to a central saccade should be minimal and irrelevant given that there is not a stimulus in the RF nor is that location a potential saccadic target (Figure 1). However, a study by Dickinson et al. [85] found that neurons in LIP can be activated by the instruction to perform a saccade, in the absence of any spatial information. Similarly, Bennur and Gold [63] found information on perceptual decisions within neurons whose RFs did not correspond with the upcoming movement.

Our results also have implications regarding the role of LIP activity changes in directing eye movements. A common theory regarding LIP is that its activity can represent the accumulation of saccade-relevant evidence. In this notion, a saccade is initiated once activity reaches a threshold. For example, during two choice motion discrimination tasks, stimulus-dependent climbing activity has been observed as animals monitored a weak motion stimulus whose direction indicates the saccade target. While the rate at which the activity accumulated was dependent on motion strength, consistent levels of activity were observed prior to saccades irrespective of the stimulus [49],[54],[56],[86],[87]. In subsequent models, such results were explained by LIP neurons integrating the motion evidence arising from MT inputs [49],[56]. Evidence consistent with a threshold mechanism has also been observed in an LIP timing study [22]. Our results differ in two fundamental respects: activity does not accumulate over time and there is not a common activity level prior to saccades (Figure 6). Thus, increases in delay period responses prior to saccades are not a universal characteristic of LIP neurons [44]. However, our model is of the same basic form as those based on the motion-based decisions in that relevant evidence is integrated and thresholded to arrive at a decision. The critical distinction is that, unlike the motion-based decision models in which the evidence for the direction of motion is present in MT neurons and evaluated by LIP neurons, in our model, evidence for the passage of time is present in LIP neurons and integrated elsewhere to arrive at a decision.

In any case, the observation that firing rates in LIP are dependent on task design is evidenced by the difference in our population between mapping, memory-guided, and self-timed saccades (Figure 4C,D). This demonstrates that LIP activity can only be interpreted with knowledge of the behavioral context [44],[63],[88]. For example, LIP activity cannot be strictly interpreted as reflecting an evidence signal whose magnitude is associated with the increasing likelihood of reaching a decision to saccade, since in our experiments activity decreases with the passage of time, which is the sole evidence the animal can use to make a saccade. Similarly, our data are not consistent with LIP solely representing an attention signal, since there are no stimulus cues present or relevant for saccades, and the observation of positive correlations between activity and interval means more activity can actually delay a saccade.

Our results also constrain the spatial distribution of timing signals within the brain. Two traditional theories concerning where timing signals originate are the central and distributed timing mechanisms [1],[89]. In the central timing model, a specific brain region produces a timing signal that is utilized for all timing-related events for all modalities. The distributed timing model suggests that there is no dedicated timing system but that the ability to represent time is an intrinsic property of distributed cell populations that are required for a given task. If LIP activity strictly reflected a broad timing system (like those described by centralized timing models), its activity would have a consistent relationship with time irrespective of saccade direction.

Because activity patterns and behavioral correlations depend in a number of respects on the particular planned saccade, our results support the notion that local neuronal populations are responsible for temporal production. First, the activity immediately preceding central and peripheral saccades is different when sorted by intersaccadic interval. Prior to central saccades, there is no evidence for a response threshold because different rates are seen at saccade onset (Figure 6A). By contrast, a common activity level is observed at peripheral saccade onset. Second, although activity was consistently predictive of saccadic interval duration, the exact relationship was significantly different for peripheral and central saccades. Activity prior to saccades made to the peripheral target was negatively correlated with interval production, while activity prior to saccades to the central target had a positive correlation.

Our results also provide insight concerning the neural mechanisms underlying timing. Multiple mechanisms have been proposed to underlie behavioral timing. Three mechanisms include the clock (pacemaker/accumulator) model, labeled lines, and population clocks [1]. In the clock model, a neural pacemaker produces rhythmic pulses. These pulses are then counted (or accumulated) in order to time an event. Clock models are generally classified as centralized systems, as this one clock is used in all timed events [1]. Because the relationship between LIP activity and behavior varies depending on the impending saccadic target, it is not consistent with a single universal representation of time. Moreover, because activity was observed to decrease rather than increase over time, accumulation as a single timing mechanism is ruled out. In the labeled line model, different neurons within a population respond at different interval lengths. For example, one neuron may respond at 100 ms while a second neuron responds at 200 ms. Labeled line models could work in a distributed timing system. For example, the labeled line population could be used to determine time according to the subset of neurons that are active. However, our data did not show strong evidence of individual neurons being significantly correlated to specific intervals.

The population clock model encodes time through the population activity of a network of neurons. There is no specific time at which neurons are active, instead dynamic interactions or time-dependent changes between neurons within the network provide information about lapsed time (e.g., short-term synaptic plasticity, inhibitory feedback, etc.). Such a model could account, in part, for the small correlation values we observed between neuronal activity and interval length if the neuronal populations underlying timed behavior were much larger than our sample. In this model, individual neurons will not contain large amounts of temporal information, consistent with the low number of individual cells that display significance in the regression and correlation analyses. From this model, we would predict that as we sampled more neurons from this population, the population correlation values would increase.

The fact that activity fluctuations are correlated to timed interval production for both saccades toward the RF and away from the RF, but those correlations are opposite in sign, suggests that activity differences between the two hemispheres may drive temporal production in our task via a push-pull mechanism. While “push-pull” models have been invoked in the past in the context of sequential saccades [90], our model relies on the specific and consistent dynamics of the push and pull signals suggested by the linear changes in firing rates over the intersaccadic intervals we have observed. The success of a simple version of such a model, a differencing of the integrated activity between hemispheres, in explaining the quantitative relationship between activity and interval supports such a proposal. Although this model does utilize climbing activity and a threshold, the location at which the push and pull signals are integrated and compared in order to signal a saccade is downstream of LIP. Therefore, LIP activity does not represent the evolving intent to make a saccade but rather provides a measure of the time elapsed since the last saccade to a particular location. Although activity from other areas, such as the frontal eye fields, could contribute to opponent push-pull fixation-saccade signals such as we propose, the mere presence of opponent signals is not sufficient to explain the behavioral timing we observed. As demonstrated by our model, the signals must vary over time in a very specific and consistent manner in order to produce precise timing.

Combined with the behavioral and physiological evidence of a reset upon every interval, our results support the notion of LIP activity providing clock-like inputs to a subsequent decision mechanism. The decaying activity that we observed is ideally suited for the accurate representation of time. First, the activity is accurately “reset” by the vigorous sensory response evoked by the saccade-driven appearance of a target within the response field (Figure 5). Second, activity decays at a constant linear rate, so that for any increment in time a common decrement in response is observed (Figure 4). Finally, perturbations in activity, which might be seen as a clock skipping a beat, have an equal effect on the eventual behavior no matter when in the interval they occur (Figure 6). The success of this simple model demonstrates that decaying activity within neuronal populations is sufficiently accurate to explain timed behaviors on the scale of seconds and suggests that temporal production may generally reflect competition between localized and precise timing representations.

## Materials and Methods

### Ethics Statement

All surgeries were done under aseptic conditions and full anesthesia in accordance with the animal care guidelines of the University of Minnesota and the National Institutes of Health.

### Task Training and Design

Two male monkeys (*Macaca mulatta*) (8.3 and 9.3 Kg) were seated in a darkened room in front of a computer monitor. Animal training began by learning to maintain fixation (within 3 deg) of a single target in order to receive a juice reward. The length of the fixation requirement was gradually increased to 1,000 ms. Once the animal could fixate for a full second, a second target appeared and the initial target was extinguished. Animals then had to fixate on one of the two targets as each was displayed for 1,000 ms. Each target was identical in size (0.15 deg) and color (white). The number of fixations (and therefore the number of timed-delayed saccades) required was gradually increased and randomized (2–10 saccades). To this point, we have somewhat mimicked previous designs, in that temporal measurement (the regularity of the fixation target disappearance and cue appearance) and temporal production (the regularity of saccades) were both likely to be occurring. To encourage the animal to rely solely upon temporal production signals, the luminance of the nonfixated target was gradually increased until both targets remained on constantly and with equal luminance throughout the trial.

Upon isolating a neuron, we recorded responses while the monkey performed a delayed-saccade task to a variety of locations. In this “mapping” task, targets were randomly sampled from eight different locations fixed radially about a mapping center (typically 10–14 deg eccentricity) at a distance of 4 deg. The animal performed single-saccade trials in which he was required to saccade to the peripheral target after waiting 1 s (Figure 1C). The location with the maximum visually evoked response was defined as the neuron's response field (RF) and used as the peripheral position in all subsequent tests. Memory trials were saved for 39 out of the 100 cells that displayed LIP activity.

Animals then performed a memory-guided delayed-saccade task [36],[37] to this RF location. The memory-guided task was used to determine which cells displayed stereotyped LIP firing activity. Trials began by requiring the monkey to fixate on a target near the center of the monitor (Figure 1D). Following fixation, a target within the RF was flashed for a brief period of time (200 ms) and then extinguished. The monkey was required to remember the location of the flashed target, while maintaining fixation at the center target. The monkey was then required to make a single saccade to the remembered location following extinction of the central target 1,000 ms after the trial began in order to receive a juice reward. To be selected for further analysis, neurons had to display a light-sensitive response to the target flashed in the RF [34],[41]–[43] (*N* = 100).

The animals then performed a self-timed rhythmic-saccade task. Trials began with the monkey fixating the first of two targets to appear on the monitor (Figure 1A). One of the targets was positioned near the center of the screen while the other target was positioned peripherally within the response field (RF) [33]–[35],[43],[91]–[93] of the neuron being recorded (dashed box in Figure 1A). Immediately following fixation of the first target (“Initial Target”), the second target appeared on the monitor (“Subsequent Target”). Once fixation of the initial target occurred, the monkey was required to perform saccades back and forth between the two targets so that a saccade occurred each second (“Self-Timed Saccades”) (±200 ms) (0.5 Hz). After both targets had appeared, no further changes in visual stimuli occurred. The monkey was required to continue making saccades (2–10) between the two targets at the 1 s interval for a randomized trial length before receiving a juice reward (Figure 1A,B). Trials randomly alternated between having the initial target appear at the central and peripheral locations to ensure that we observed both saccadic directions for each interval within the sequence.

Saccade targets were constantly displayed following the first interval of the trial in order to minimize sensory anticipation [23]. To help minimize reward expectations, the hazard function, which represents the instantaneous probability of the trial ending given that it has not yet ended, was flat throughout each trial [16],[94]. Therefore, the instantaneous probability that the animal received a reward at any given instant was kept constant throughout each trial. Trial times were randomly chosen from an exponential distribution (decay constant = 1,000 ms). Trials could end at any point, including the middle of an interval, in order to further dissociate saccadic movements from reward. Average trial length for both animals equaled 3.96 s (SD = 1.14 s). A minimum of 50 saccades was required from each cell while the animal performed the self-timed task.

Eye movements made to the peripheral target (in the direction of the RF) are termed “peripheral” saccades, while eye movements made to the central target (or away from the RF) are termed “central” saccades (Figure 1A). For clarity, during this task, when the animal was fixating on the central target, the response field was located at the peripheral target. The animal's next saccade would then be made to the peripheral target, toward the RF. However, once the animal fixated on the peripheral target, the RF was no longer located at either target and the next move (central saccade) would be made away from the direction of the RF.

Although the delay period of the rhythmic and memory tasks remained the same (1,000 ms), the self-timed rhythmic-saccade task differed from the memory-guided delayed-saccade task in a number of ways (Figure 1A,D). First, in the memory task, the peripheral target was extinguished after its initial appearance within the neuron's response field instead of remaining throughout the entire trial. Thus, the animal was required to remember the target location following the disappearance of the peripheral target. Second, the memory task always required just a single saccadic movement and rewards were always delivered immediately following a correct saccade. Lastly, the animal was cued when to make the movement by the extinction of the central fixation point during the memory task. Because the animal could rely solely on this external cue to initiate a saccade to that location, no temporal production signals were required for successful completion of the memory task.

### Electrophysiology

Prior to training, animals were chronically implanted with titanium head posts in order to stabilize head position. Animals were also implanted with scleral eye coils in order to monitor eye position (sampling rate of 200 Hz), although an infrared eye tracking system (iView X Hi-Speed Primate camera system, SensoMotoric Instruments) was used most often to track eye position. Following training, animals were implanted with chronic stainless steel or customized PEEK (polyether ether ketone) recording cylinders. Cylinders were placed, stereotactically, in a manner that allowed electrode penetration of the lateral bank of the intraparietal sulcus (area LIP). Area LIP was identified anatomically using MRI prior to recording cylinder implantation. Co-registered MRI and CT images taken after chamber placement were used to confirm electrode placement within LIP (Figure 3A,B). All surgeries were done in accordance with animal care guidelines of the University of Minnesota and the National Institutes of Health. Surgeries were performed under aseptic conditions with full anesthesia.

Single-cell recordings were done from 175 well-isolated neurons using standard extracellular recording techniques (Mini-Matrix, Thomas Recording). Action potentials were isolated on the basis of waveform (APC, FHC), sampled (1,000 Hz), and digitized for on and off-line analysis. One hundred of the 175 neurons sampled displayed both the light-sensitive and the non-zero memory activity during the memory task and they are analyzed here. Recordings were typically taken from the right hemisphere (26/50 cells for animal 1 and 50/50 cells for animal 2).

### Data Analysis

Visual stimulation, behavioral control, and data acquisition were controlled using customized computer software (http://www.ghoselab.cmrr.umn.edu/software.html). Online analyses of average firing rate were used to determine RF locations. Offline analyses of firing rates, correlations (corrcoef), and regressions (regress) relative to the events of the saccade tasks were done using Matlab (MathWorks). Average firing rates were smoothed by convolving with a Gaussian kernel (SD = 35 ms). However, no smoothing was done for any correlation analysis. Saccade onset was defined according to eye velocity (>85 deg_/s) in conjunction with the computer software's recognition of the animal's gaze arriving within the fixation window. Intersaccade times are defined as the interval between successive saccades. The first saccadic intervals of a trial were analyzed separately from all subsequent intervals since, for initial saccades to the central position, the appearance of the subsequent target within a neuron's RF elicited a response. Significant increases in presaccadic activity were calculated by comparing firing activity within 150 ms of saccade initiation (−150 to 0) with the activity 250 ms prior to that interval (−400 to −150) (*t* test, *p*<0.05).

In order to determine what factors are associated with firing rate changes, we generated a prediction of neural activity by convolving observed neural activity aligned with one saccade direction with the intersaccade distribution times aligned with the other saccade direction. For example, if we convolve the intersaccade distribution times aligned to central saccades with the actual firing rate aligned to peripheral saccades, we get a prediction for central saccade aligned activity (see Figure 5A for example) [convolution: (*f* *d)(t) = ∫ f(τ)d(t−τ)dτ]. The difference between this prediction and the observed firing rate for activity aligned to central saccades indicates how well activity associated with peripheral saccades can completely explain task-related modulations in activity. The same analysis is then repeated using activity aligned to central saccades. Fit is given by: *%Fit = (1* ***−*** NMSE)*100, where NMSE is the normalized mean squared error. The NMSE is calculated by dividing the mean squared error by the explainable variance of the actual unsmoothed firing rate (variance of firing rate over the interval − average variance of all time points of the rate).

## Supporting Information

Figure S1 Perisaccadic firing activity segregated by saccade sequence and animal. (A) Firing activity aligned to central saccades and segregated based on saccade number within a trial for animal 1. (B) Same as in (A) except for animal 2. (C and D) Same as in (A and B), respectively, except aligned to peripheral saccades. Saccade intervals: orange, 2nd saccade; green, 3rd; magenta, 4th; black, 5th. Colored bars along the *x*-axis indicate fixation location (blue, central target; red, peripheral target). Only the first five sequences with static targets throughout the intervals are shown (first sequences not shown). Only data points with greater than 15 trials are shown (see black trace in B).
(EPS)

## Abbreviations

CT: computed tomography
LIP: lateral intraparietal area
MI: memory index
MRI: magnetic resonance imaging
MT: medial temporal area
RF: response field
